# Absence of hexavalent chromium in marine carbonates: implications for chromium isotopes as paleoenvironment proxy

**DOI:** 10.1093/nsr/nwaa090

**Published:** 2020-05-08

**Authors:** Ziyao Fang, Liping Qin, Wei Liu, Tao Yao, Xiaoyan Chen, Shiqiang Wei

**Affiliations:** CAS Key Laboratory of Crust-Mantle Materials and Environments, University of Science and Technology of China, Hefei 230026, China; CAS Center for Excellence in Comparative Planetology, Hefei 230026, China; CAS Key Laboratory of Crust-Mantle Materials and Environments, University of Science and Technology of China, Hefei 230026, China; CAS Center for Excellence in Comparative Planetology, Hefei 230026, China; National Synchrotron Radiation Laboratory, University of Science and Technology of China, Hefei 230029, China; National Synchrotron Radiation Laboratory, University of Science and Technology of China, Hefei 230029, China; CAS Key Laboratory of Crust-Mantle Materials and Environments, University of Science and Technology of China, Hefei 230026, China; National Synchrotron Radiation Laboratory, University of Science and Technology of China, Hefei 230029, China

**Keywords:** Cr isotopes, XANES, speciation, atmospheric oxygen level

## Abstract

The oxygenation of Earth's atmosphere is widely regarded to have played an important role in early-life evolution. Chromium (Cr) isotopes recorded in sedimentary rocks have been used to constrain the atmospheric oxygen level (AOL) over geological times based on the fact that a positive Cr isotopic signature is linked to the presence of Cr(VI) as a result of oxidative continental weathering. However, there is no direct evidence of the presence of Cr(VI) in sedimentary rocks yet. Carbonates are most widely distributed over geological times and were thought to have incorporated Cr(VI) directly from seawater. Here, we present results of Cr valence states in carbonates which show Cr(III) is the dominant species in all samples spanning a wide range of geological times. These findings indicate that Cr(VI) in seawater was reduced either before or after carbonate precipitation, which might have caused Cr isotopic fractionation between seawater and carbonates, or marine carbonates preferentially uptake Cr(III) from seawater. As Cr(III) can come from non-redox Cr cycling, which also can cause isotopic fractionation, we suggest that positively fractionated Cr isotopic values do not necessarily correspond to the rise in AOL.

## INTRODUCTION

Several lines of evidence have suggested that atmospheric oxygen concentrations first rose during the Great Oxygenation Event (GOE) approximately 2.4 billion years ago and a second increase that likely began in the later Neoproterozoic, known as the Neoproterozoic Oxygenation Event (NOE) [1]. However, some geochemical data have suggested that transient rises in the atmospheric oxygen level (AOL) occurred before and after the GOE as a result of microbial oxygen-producing photosynthesis [2,3].

Chromium (Cr) isotopes in sedimentary rocks are an emerging tool for tracing such changes in the AOL [2–6]. The use of Cr isotopes to trace atmospheric oxygenation is based on the fact that Cr in aquatic systems is dominated by Cr(VI) and the reduced form Cr(III), which dominates in the silicate Earth, is relatively insoluble and less mobile [7]. The oxidation of Cr(III) in silicate Earth systems is highly dependent on the presence of manganese oxides, which are stable in relatively high-free-oxygen environments [2]. The oxidation of igneous Cr(III) and the subsequent partial reduction of dissolved Cr(VI) during transportation can cause large isotopic fractionation [7]. As a consequence, the aquatic Cr reservoir would be characterized by a positive shift in Cr-isotope ratios (reported as δ^53^Cr values) from the relatively uniform value of −0.124 ± 0.101‰ observed for igneous rocks [8]. Marine sediments could capture the Cr-isotope signal from seawater; thus, the highly fractionated positive δ^53^Cr values preserved in marine sedimentary rocks should mirror those of the contemporaneous seawater and further reflect the signal of terrestrial oxidative weathering [2].

A key point of this model is the generation of Cr(VI) in oxidative weathering during atmospheric oxygenation events. However, no direct evidence of hexavalent Cr in sedimentary rocks has yet been reported. Laboratory co-precipitation experiments revealed that CrO_4_^2−^ can directly substitute CO_3_^2−^ in a carbonate crystal lattice [9]; therefore, marine carbonates are believed to directly incorporate Cr(VI) in seawater [10], whereas other chemical sediments in which positive δ^53^Cr values have been observed, such as iron formations (IFs), ironstones and shales, are expected to contain the reduced form Cr(III). Here, we present Cr speciation in carbonates using X-ray absorption near edge structure (XANES) analysis and paired geochemical data to provide new insights into Cr incorporation in natural carbonates and the application of its isotopic composition to probe paleo-redox environment changes.

## RESULTS

Six carbonate samples ranging from ∼1.44 Ga to ∼0.83 Ma in age including one natural carbonate standard BCS-CRM513 (with an age of ∼0.35 Ga) were studied for Cr speciation, chemical composition and Cr isotopic composition (sample details are given in the Supplementary Data at NSR online). Rare earth elements and manganese/strontium (Mn/Sr) ratio results indicate that these samples were likely not influenced by meteoric diagenesis and hydrothermal alteration (Supplementary Data). Chromium concentrations in carbonate fractions of the samples range from 1.14 to 16.21 ppm, within the large variation (from the 10-ppb level to the 10-ppm level) observed in modern marine carbonates [10–12]. Because the Cr concentration in silicates is much higher than that in carbonates, a large proportion of Cr is present in the clast fraction, despite the weight percentage of carbonate fraction being at least ∼80% (Table 1 and Supplementary Table 1). However, carbonate still contains ≥12.56% of the total Cr (Table 1). The Cr isotopic compositions (δ^53^Cr) in the carbonate fraction of these samples range from –0.36‰ to 1.38‰, and three of the samples have positively fractionated Cr isotopic compositions (Table 1).

**Table 1.** Chromium concentration and isotopic composition of the carbonate samples.

Age (Ma)	Sample	Cr_carbonate_ (ppm)^a^	Cr_residue_ (ppm)^b^	Cr_carbonate_/Cr_total_ (%)	δ^53^Cr (‰)^c^
∼630	WJ705.6	2.63 ± 0.01	10.52 ± 1.05	20.00 ± 1.60	−0.36 ± 0.05
∼560	12JLW44	7.07 ± 0.04	49.24 ± 4.92	12.56 ± 1.10	0.87 ± 0.05
∼250	XK127	1.22 ± 0.01	8.04 ± 0.80	13.17 ± 1.14	−0.08 ± 0.05
∼350	BCS-CRM513	3.92 ± 0.02	2.04 ± 0.20	65.77 ± 2.23	1.38 ± 0.05
∼0.83	1460A26F1W110/116	16.21 ± 0.35	4.91 ± 0.49	76.94 ± 2.74	0.65 ± 0.05
∼1440	J1–52.6	1.14 ± 0.01	1.26 ± 0.13	47.50 ± 2.61	−0.02 ± 0.05

^a^Cr concentration in the carbonate fraction of the samples, expressed as a ratio of the Cr weight in the carbonate fraction to the whole weight of the sample, determined by the isotope-dilution method. This fraction was leached by 5% acetic acid. ^b^Cr concentration in the clast of the sample, expressed as a ratio of the Cr weight in the clast fraction to the whole weight of the sample, determined by ICP–MS. This fraction consisted of the residue after the 5% acetic-acid digestion and was fully dissolved by an acid mixture of HF + HNO_3_. ^c^δ^53^Cr value of the carbonate fraction.

**Figure 1.**
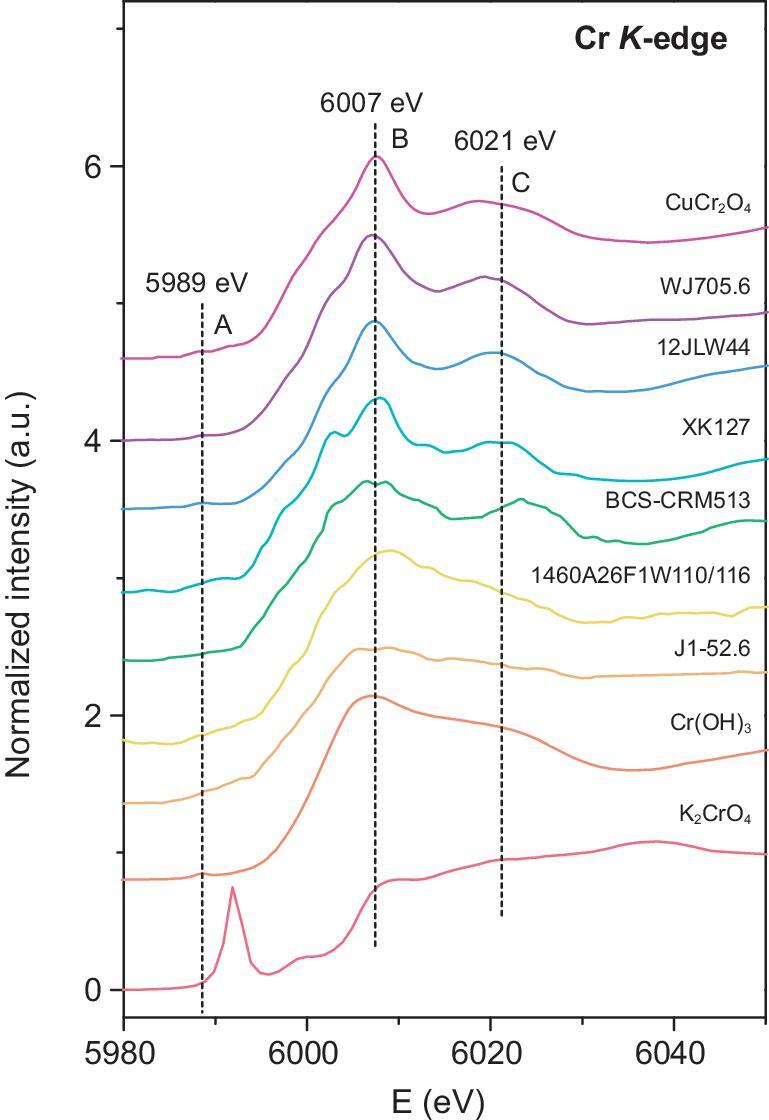
XANES spectra of the carbonate samples and the reference materials. The curves show the normalized spectra of the Cr(VI) standard K_2_CrO_4_, the Cr(III) standard Cr(OH)_3_, the Cr(III) standard CuCr_2_O_4_, Ediacaran carbonate WJ705.6 (∼0.63 Ga) from the Wangji section, Ediacaran carbonate 12JLW44 (∼0.56 Ga) from the Jiulongwan section, Triassic carbonate XK127 (∼0.25 Ga) from the Xiakou section, Carboniferous carbonate BCS-CRM513 (∼0.35 Ga) from the Derbyshire section, Quaternary carbonate 1460A26F1W110/116 (∼0.83 Ma) from the IODP site U1460 and Mid-Proterozoic carbonate J1–52.6 (∼1.44 Ga) from the Yanshan section. The spectra of the natural carbonate samples are smoothed with a three-iteration moving average because of the relatively poor signal-to-noise ratio. A, B and C denote the pre-edge features, edge crests and post-edge peaks, respectively.

**Figure 2.**
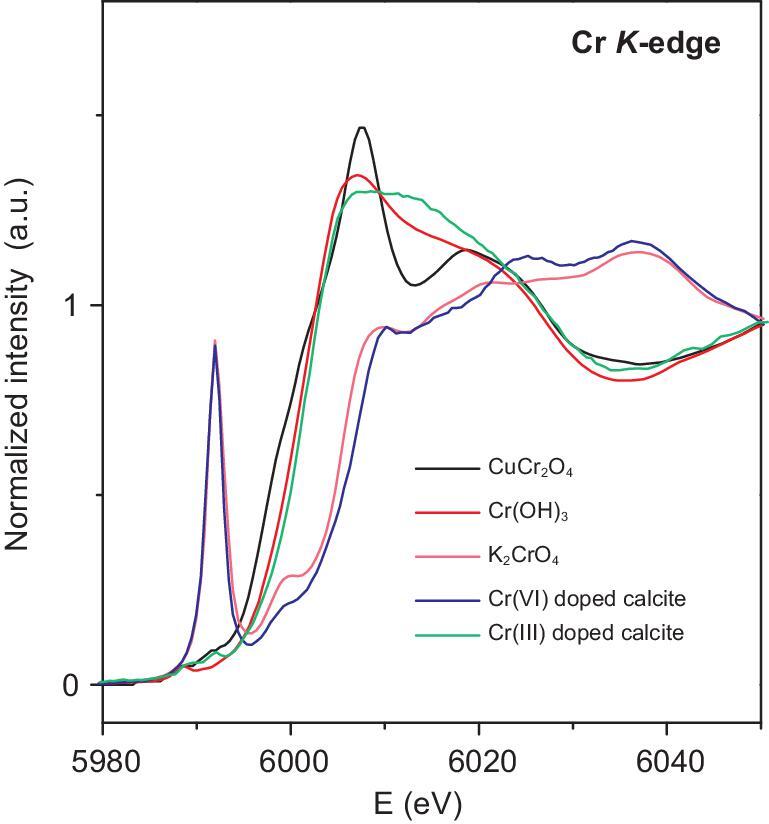
Normalized XANES spectra of the Cr(VI) standard K_2_CrO_4_, the Cr(III) standard CuCr_2_O_4_ and Cr(OH)_3_ and two synthetic calcite samples co-precipitated with CrO_4_^2−^ or Cr^3+^, respectively. The pre-edge peaks of the synthetic samples are almost the same as the standards, indicating that the pre-edge feature of the XANES spectra is a robust evaluation criterion of Cr valence regardless of the specific coordination environment and sample matrix.

XANES spectra are used to distinguish different Cr valence states in carbonates based on the significant difference in spectra features between Cr(III) and Cr(VI). A prominent peak at the pre-edge region occurs when Cr is present as non-centrosymmetric tetrahedral Cr(VI), owing to the electronic transition from the 1s orbital to the empty 3d orbital. This transition is virtually forbidden for Cr(III), which always occurs as a centrosymmetric octahedral Cr(III)O_6_ structure [9,13]. In addition, the energy of the edge crest for Cr(III) is lower than that for Cr(VI) [9]. These criteria can help to determine the valence of Cr in samples. The XANES results are shown in Fig. 1. Because only bulk sample powder can be measured, the spectra should show the characteristics of the total Cr in the carbonate fraction and the clast fraction. Despite that, the height of the pre-edge peak for Cr(VI) should be linearly proportional to the Cr(VI)/Cr_total_ ratio in the bulk sample, regardless of the specific coordination environment and sample matrix (e.g. Fig. 2) [13]. For all our samples, carbonate contains ≥12.56% of the total Cr. Therefore, if Cr(VI) is a major form in the carbonate fraction, the spectra should have a resolvable pre-edge peak, even though the clast contains a large fraction of Cr in the samples. In addition, all of the Cr(VI) signal should come from the carbonate fraction as the clast is expected to contain only Cr(III). However, the features of all of the spectra of the measured carbonate samples are similar to those of the Cr(III) standard [Cr(OH)_3_ or CuCr_2_O_4_], indicating a very small, if any, contribution of Cr(VI) to the total Cr in these samples. Specifically, it is clear that the pre-edge feature, the edge crest and the post-edge peak of the WJ705.6 (∼0.63 Ga) and 12JLW44 (∼0.56 Ga) samples all resemble that of the CuCr_2_O_4_ standard, demonstrating that almost all of the Cr in these three samples exists as Cr(III). For the other four samples, the edge crest and the post-edge peak are similar to those of CuCr_2_O_4_ or Cr(OH)_3_, but the absorption intensity increases slightly after ∼5989 eV, possibly indicative of a very small fraction of Cr(VI). However, these features were likely caused by the poor signal-to-noise ratio of the spectra. For BCS-CRM513 (∼0.35 Ga), 1460A26F1W110/116 (∼0.83 Ma) and J1–52.6 (∼1.44 Ga), the Cr_carbonate_/Cr_total_ is over ∼50%, suggesting that Cr(III) dominates the carbonate fraction even if Cr(VI) is present. However, for XK127 (∼0.25 Ga), it is difficult to assess the predominant Cr species of the carbonate fraction because the Cr_carbonate_/Cr_total_ is only 13.17% in this sample.

## DISCUSSION

The deposition times of the samples covered the Mid-Proterozoic, when oxidative Cr cycling is thought to have been inhibited, and the transition through the Neoproterozoic to the Phanerozoic, when Cr(VI) is thought to have dominated Cr cycling [14]. However, the Cr valence state results showed that Cr(III) is the dominant Cr species in carbonates regardless of the δ^53^Cr value and the age of the sample, in contrast to the previous view based on laboratory experiments that chromate in seawater would be directly incorporated into the calcite crystal lattice [9]. It is possible that terrestrial Cr(VI) was reduced in seawater before carbonate precipitation during oceanic anoxia events, such as in XK127 (∼0.25 Ga), which was deposited in the slope environment. However, for three samples [12JLW44 (∼0.56 Ga), BCS-CRM513 (∼0.35 Ga) and 1460A26F1W110/116 (∼0.83 Ma)] deposited in the continental shelves possibly under an oxic environment and characterized by positive δ^53^Cr values, if Cr isotopes in carbonates indeed record oxidative Cr weathering to some extent and the primary species of Cr in seawater is hexavalent Cr, reduction of Cr(VI) is to occur during or after carbonate formation. Two possible mechanisms can contribute to this reduction. First, because no abiotic reduction of Cr(VI) was observed during a co-precipitation experiment of CrO_4_^2−^ with calcite [9], we consider the possibility of microbial reduction. Carbonate precipitation is often associated with high primary production by phytoplankton. Previous work has shown that the Cr(III)/Cr_total_ value of seawater increases strongly with bacterial biomass and marine primary productivity, suggesting large-scale photoreduction of Cr(VI) [15,16]. Furthermore, some carbonates consist directly of biologic skeletons (e.g. corals, foraminifera) and it has been suggested that marine phytoplankton preferentially uptake Cr(III) in seawater [17]. Recent studies reported that the δ^53^Cr values of marine biogenic carbonates worldwide are systematically lower than those of the coexisting seawater, which might be a result of Cr(VI) bio-reduction [11,18]. However, biogenic carbonates tend to have very low Cr concentrations (<0.1 ppm for corals; <0.5 ppm for most foraminifera and molluscan shells) [11,18,19], but all of the studied carbonate rocks had 1.14–16.21 ppm Cr in the carbonate fraction; thus, the direct contribution of Cr in skeletons to the carbonate rocks is expected to be small. Therefore, Cr bio-reduction before or during carbonate precipitation may not be the main reason for the absence of Cr(VI) in our carbonate samples.

**Figure 3.**
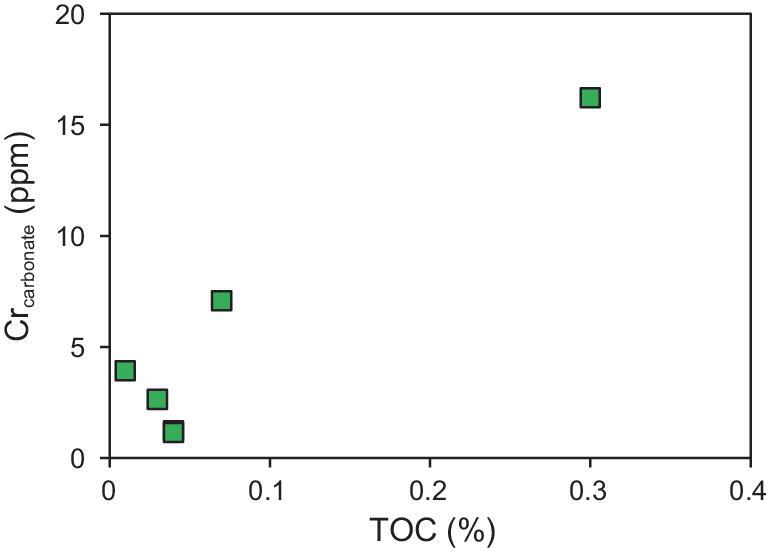
Plot of Cr concentrations in carbonate fraction versus TOC values in bulk samples.

Alternatively, post-depositional processes might account for the absence of Cr(VI) in carbonates. Our samples were not altered by hydrothermal or meteoric fluids, but the influence from pore water during early diagenesis should not be neglected. A recent study showed that Cr in sedimentary foraminifera could be mainly post-depositional and come from bottom/pore water [20]. A similar process could also influence non-skeletal carbonates. In this case, most Cr in carbonates might be directly taken up in pore water and have been previously reduced in the relatively anoxic environment driven by the microbial decomposition of organic matter. This is supported by the plot of Cr concentrations in carbonate fraction versus the total organic carbon (TOC) values of our samples (Fig. 3), which shows a plausible positive correlation and may suggest that organic matter promotes the reduction of Cr(VI) and then the incorporation of Cr(III) into the carbonate. Besides, studies on pore water have suggested that metastable aragonite and Mg-calcite can be influenced by dissolution and recrystallization [21], thus Cr(VI) in the crystal might be released into the pore water and be reduced.

In either of the cases discussed above, the reduction of Cr(VI) is likely not quantitative in the relatively oxic carbonate-deposition environment, and as a result, the carbonates might not faithfully record the Cr isotopic composition of the contemporaneous seawater. Studies on modern marine carbonates and paired local seawater indicate that carbonates have systematically lower δ^53^Cr values relative to local seawater [12,18]. A recent study conducted co-precipitation experiments which showed that Cr(VI) incorporated in calcite is enriched in light Cr isotopes and the isotopic fractionation is pH-dependent, which can explain the isotopic bias between natural marine carbonates and local seawater [22]. However, our results that Cr(III) dominates in natural marine carbonates indicate that the isotopic variations between carbonates and seawater are more likely related to redox processes, instead of the pH of the forming fluid.

**Figure 4.**
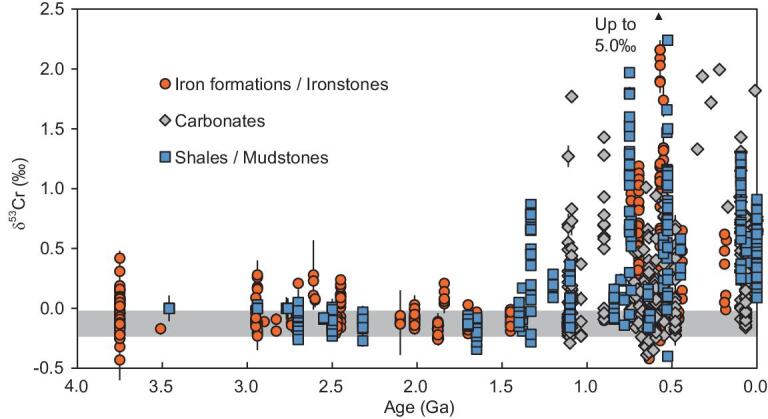
Cr-isotope data of sedimentary rocks throughout Earth history from the literature. Red circles represent the data for IFs or ironstones [2,3,14,30–36]; blue squares denote data for shales, mudstones or marine sediments [4,14,37–43]; gray diamonds denote data for carbonates [5,6,10,12,44–49]. The gray band indicates the range for bulk silicate Earth (BSE) [8].

The discussions above assume that Cr in carbonates ultimately come from Cr(VI) in seawater, which is the dominant Cr species in the oxic ocean. However, Cr(III) is ubiquitous even in modern river water and oceans, and can sometimes be the dominant species [23,24]. Cr(III) is more particle-reactive and may have higher affinity with carbonates compared with Cr(VI). Therefore, it is possible that some carbonates preferentially directly uptake the Cr(III) species in seawater, even when the dominant Cr species in seawater is Cr(VI). In natural aquatic environments, Cr(III) can result from the reduction of Cr(VI), but can also come from the non-redox weathering of Cr-bearing source rocks induced by CO_2_ or organic acid [23,25], especially during the early geological history when the AOL was low [26]. One piece of evidence is suggested by the presence of Cr(III) in J1–52.6 (∼1.44 Ga), which was deposited at a time when oxidative Cr cycling is thought to have been inhibited, but Cr has to be mobilized into aqueous systems before they can be incorporated into the sediments [14]. Thus, Cr isotopic compositions of carbonates might record the signal from Cr(III) in seawater, some of which could come from non-redox weathering. It has been found that ligand-promoted dissolution of Cr(III) (hydroxide, chromitite and silicate rocks) could be accompanied by Cr isotopic fractionation, with heavier isotopes preferentially liberated [27,28]. Therefore, positive δ^53^Cr values in carbonates might not necessarily represent the presence of oxidative Cr weathering. Despite that, the fractionation magnitude of the valence change between Cr(VI) and Cr(III) is much greater than that for ligand-promoted dissolution [7,27,28]. Hence, we infer that the significantly positively fractionated Cr isotopic composition observed in some sediments can be readily explained by oxidative weathering (Fig. 4), but for slightly positively fractionated δ^53^Cr values, it may not reflect atmospheric oxygenation events.

## CONCLUSION

This study reports the first paired Cr valence state and Cr isotopic composition measurements of marine carbonates from a wide range of geological times. The major conclusions are as follows:

- XANES results show that Cr(III) dominates in all carbonates regardless of the deposition ages and their Cr isotopic compositions. This is in apparent contrast with a previous model which suggested that Cr(VI) in seawater could directly occupy the carbonate crystal lattice and be preserved.
- A possible explanation for the absence of Cr(VI) in some carbonates is that Cr(VI) in seawater might have been reduced by microbes during carbonate precipitation or reduced by pore water after precipitation. In both cases, the reduction of Cr(VI) is likely not quantitative, which could explain the observed systematically lower δ^53^Cr values for modern marine carbonates relative to local seawater.
- Another possible explanation is that carbonates preferentially directly uptake Cr(III) in seawater. This is likely the reason for the absence of Cr(VI) in J1–52.6 (∼1.44 Ga) and possibly for some other samples as well. Cr(III) could come from the non-redox weathering of Cr. As non-redox Cr cycling also can cause isotopic fractionation, the slightly positively fractionated Cr isotopic values previously reported for some sedimentary carbonates do not necessarily correspond to the rise in the AOL as previously suggested.

Both mechanisms will affect the use of the Cr isotopic composition of marine carbonates to trace the redox condition of the contemporaneous seawater and atmosphere. It is likely that a different mechanism dominates at different times and in different regions. To further understand the Cr valence state in sedimentary carbonates and Cr cycling, more samples deposited at different geological times and in different marine environments need to be examined, especially those least affected by diagenesis. In addition, carbonate co-precipitation experiments need to be carried with Cr(III) to understand the possible incorporation mechanism of Cr(III) into carbonate.

## METHODS

### XANES measurement

XANES spectra were collected at beamline 1W1B of the Beijing Synchrotron Radiation Facility and beamline BL14W1 of the Shanghai Synchrotron Radiation Facility in China. We selected four Cr compounds as reference materials: Cr metal (Cr^0^), Cr(OH)_3_ (Cr^3+^), CuCr_2_O_4_ (Cr^3+^) and K_2_CrO_4_ (Cr^6+^). Another two synthetic carbonate samples co-precipitated with Cr(VI) or Cr(III) were also determined for comparison. Data for CuCr_2_O_4_ were obtained from the XAFS database (xafs.org). The spectrum of the Cr metal was collected in transmission mode at room temperature using a gas-filled ionization chamber. The first peak of this derivative spectrum was set at 5989 eV for energy calibration. The other two reference materials were diluted to 1 wt%(Cr) with graphite powder and tabletted; spectra were collected in fluorescence mode at room temperature using a Lytle detector positioned orthogonal to the X-ray beam. Owing to the extremely low Cr content in the natural carbonate samples, a Ge solid-state detector was used for fluorescence detection. In order to improve the signal-to-noise ratio, we increased the integration time for every energy step to 6 s and every carbonate sample was measured two or three times and then the spectra were merged together. The total measurement time for each sample was up to 2 h. Even so, the signal-to-noise ratio of the spectra was still poor and the spectra of the carbonate samples were further smoothed using a three-iteration moving average. During the measurements, the energy step was set to 30 eV in the far pre-edge region, 5 eV in the near pre-edge region, 0.5 eV in the near edge region, 1 eV in the near post-edge region and 50 eV in the far post-edge region. Data were analysed according to standard procedures using Athena software. The spectra were normalized after energy calibration.

### Analytical procedures for determining major and trace elements and Cr isotopic composition

Carbonate samples were crushed into fine powder using a ball mill or an agate mortar. To determine the major and trace-element concentrations, ∼20 mg of sample powder was first treated with 3 mL 5% acetic acid in a centrifuge tube (4 h for limestones; ≥8 h for dolostones) to dissolve the carbonate fraction. Then, the sample was centrifuged to separate the solution from the residue. The supernatant was pipetted into a PFA Savillex™ beaker and dried on a hotplate at 130°C. Afterwards, the dried solution sample was treated with concentrated nitric acid three times at 130°C to digest the organic material and then dissolved in 2% HNO_3_ before measuring major and trace-element concentrations using an inductively coupled plasma–mass spectrometer (ICP–MS). The Cr concentration of the carbonate fraction was also determined using the isotope-dilution method during Cr isotopic measurement (see next paragraph). To determine the Cr concentration in the clast, the carbonate-free residue in the centrifuge tube was digested using HF + HNO_3_ and then measured using ICP–MS. For comparison purposes, the Cr concentration was expressed as the ratio of the Cr weight in the carbonate fraction or in the clast fraction to the whole weight of the sample.

For Cr isotopic measurement of the carbonate samples, 50–1000 mg (containing ∼1 μg Cr in the carbonate fraction) of the sample powder was first doped using an appropriate amount of ^50^Cr–^54^Cr double spike and was then treated with 6–30 mL of 5% acetic acid. A double spike was added to correct any possible mass-dependent isotopic fractionation during leaching, the chemical purification procedure and mass-spectrometer measurement. Further separation procedures are as described above. The dried sample was dissolved in 6 mol/L HCl and heated to >130°C for 2 h. Next, the sample solution was diluted to 1 mol/L HCl with MilliQ (MQ) water and subjected to cation chromatography to separate the Cr from the matrix. Briefly, 10 mL of BioRad AG 50W-X8 (200–400 mesh) cation-exchange resin was first cleaned and then conditioned using 1 mol/L HCl. The sample solution was loaded onto the column in 6 mL of 1 mol/L HCl and washed twice with 10 mL of 1 mol/L HCl. The Cr was eluted as neutral molecules and collected with the eluent. The sample was further purified using another cation-exchange column filled with 0.33 mL BioRad AG 50W-X8, with 0.5 mol/L HF and 1 mol/L HCl to elute the remaining interferences and matrix and 2 mol/L HCl to elute the Cr [29]. The blank of the total procedure was typically <5 ng and the yield was typically 70–80%.

Purified Cr samples were analysed using a Neptune Plus multiple-collector inductively coupled plasma–mass spectrometer (MC-ICP-MS). Seven Faraday cups were used to monitor the intensities of four Cr isotopes (^50^Cr, ^52^Cr, ^53^Cr, ^54^Cr), as well as ^49^Ti, ^51^V and ^56^Fe to correct for the interference on ^54^Cr from ^54^Fe and that on ^50^Cr from ^50^Ti and ^50^V. Measurements were performed in medium- to high-resolution mode to minimize polyatomic interferences such as ^40^Ar^12^C, ^40^Ar^14^N and ^40^Ar^16^O. The typical intensity of the ^52^Cr beam was 4–8 V. Each measurement consisted of 4 blocks with 30 cycles of isotopic ratios, with each cycle integrating the beam intensity for 8.389 seconds. The spiked internal standard (SCP) and the spiked National Institute of Standards and Technology (NIST) standard reference material (SRM) 3112a were analysed at the beginning of each analytical session to ensure instrumental accuracy. The spiked SCP was analysed at intervals of four or five samples during each session. Each sample was analysed twice. The detailed analytical procedure is described in ref. [29]. Chromium-isotope data are expressed as the relative deviation from the NIST SRM 979:

$$\begin{eqnarray*}{\delta ^{53}}{\rm{Cr}} &=& \big[ {{\left( {^{53}{\rm{Cr}}{/^{52}}{\rm{Cr}}} \right)}_{{\rm{sample}}}}/\nonumber\\&&{{\left( {^{53}{\rm{Cr}}{/^{52}}{\rm{Cr}}} \right)}_{{\rm{SRM9}}79}} - {\rm{ }}1 \big]{\rm{ }} \times 1000.\end{eqnarray*}$$

The long-term reproducibility of an internal standard solution (SCP) was better than 0.05‰. The measured δ^53^Cr value of the carbonate standard material (BCS-CRM513) was in agreement with published data (Table 1) [10,12].

## Supplementary Material

nwaa090_Supplemental_File
