# Supplementary material for: Absence of hexavalent chromium in marine carbonates: implications for chromium isotopes as paleoenvironment proxy
Source: Natl Sci Rev. 2020 May 8;8(3):nwaa090. doi: 10.1093/nsr/nwaa090 (PMC8288429; doi:10.1093/nsr/nwaa090)
Supplement: nwaa090_Supplemental_File [file nwaa090_supplemental_file.docx]

Supplementary Data for

**Absence of hexavalent chromium in marine carbonates: implications for chromium isotopes as paleoenvironment proxy**

Ziyao Fang, Liping Qin, Wei Liu, Tao Yao, Xiaoyan Chen and Shiqiang Wei

**This file includes:**

Samples

Supplementary text

Supplementary Figure 1

Supplementary Table 1

Supplementary references

**Samples**

The ~1.44 Ga Mid-Proterozoic sample J1-52.6 from the Tieling formation

Sample J1-52.6 is a micritic dolomite in the Tieling formation that was collected from a drill core located in Beizhangzi Village, Kuancheng Town, Hebei Province, North China. The recently determined SHRIMP U-Pb zircon age of a K-bentonite bed in the Tieling formation is 1437 ± 21 Ma [1], nearly the end of the Calymmian Period. The atmospheric oxygen level (AOL) in the Mid-Proterozoic is thought to have been insufficient to stabilize manganese oxide, so oxidative weathering of terrestrial trivalent Cr was inhibited [2]. However, a recent study showed that significantly positively fractionated Cr isotopes were in place at ~1.33 Ga [3]. Geochemical analyses of shales from the Xiamaling formation overlaying the Tieling formation indicated that a probable oxygenation event might have occurred at ~1.40 Ga [4], but controversy remains [5]. Until now, no direct geochemical evidence about the redox conditions of the Tieling formation has been reported. The δ^53^Cr value of J1-52.6 (-0.02 ± 0.05‰) lies in the range of igneous reservoir, indicating low levels of oxygen, based on the previous Cr cycling model [6].

The ~0.63 Ga (WJ705.6) and ~0.56 Ga (12JLW44) Ediacaran samples in the Doushantuo formation

Two carbonate samples from two sections in the Doushantuo formation were used in this study. WJ705.6 was collected from a drill core west of Wangji Town, Hubei Province, South China, and 12JLW44 was collected from the Jiulongwan section about 10 km east of Zigui City, Hubei Province, South China [7]. The U-Pb zircon ages of the volcanic ash beds at the bottom and top of the Doushantuo formation are 635.2 ± 0.6 Ma and 551.1 ± 0.7 Ma [8], respectively, covering ~90% of the Ediacaran period. WJ705.6 is from the bottom dolomite overlaying the Nantuo tillite, and 12JLW44 is a limestone from the lower part of the interval corresponding to the “Shuram anomaly” of carbon isotopes. A recent rock magnetic chronostratigraphy study of the duration of the Shuram anomaly suggested that the onset of the Shuram anomaly occurred at ~560 Ma [9], which might be slightly earlier than the deposit time of sample 12JLW44. Positively fractionated Cr isotopes in the IFs and shales occurred in the Neoproterozoic at ~750 Ma [2, 6], and some researchers argue that the oxidative Cr cycle has been in place since that time. Recent observations of positively fractionated Cr isotopes in shales and carbonates suggest that the time of the oxidation event might be earlier (~1.33 Ga) [3, 10]. A model of Ediacaran ocean chemistry comprises a redox-stratified ocean consisting of oxic shallow water in the continental shelf, a metastable zone of anoxic and sulfidic water in the slope facies, and ferruginous deep ocean water [11]. The Doushantuo formation in the Wangji section and the Jiulongwan section deposited in the continent-ward inner shelf area below or near the wave base [7, 12], where the ocean redox condition is expected to be oxic, so that if oxidative weathering of terrestrial Cr(III) did occur, the Cr(VI) in the shallow seawater could be preserved in the carbonate.

The ~0.35 Ga Lower Carboniferous carbonate standard sample BCS-CRM513

Sample BCS-CRM513 is a limestone standard material from the Bureau of Analysed Samples Ltd. It was collected from the Longcliffe Quarry, north of Derbyshire, UK. The limestone widely distributed in Derbyshire is thought to have formed at the Avonian age, equal to the lower part of the Mississippian series, Lower Carboniferous, in the international geologic timescale [13]. The depositional environment was a marine shelf [13]. It is widely accepted that the AOL rose to the present-day level after the Neoproterozoic Oxygenation Event [14]. Geochemical data indicate that oxygen levels also varied appreciably in the Phanerozoic [15], reached similar level to the present during the Lower Carboniferous, and experienced further increase during the Carboniferous [15]. Theoretical calculations based on rates of Mn oxidation showed that the minimum O_2_ level required to achieve Cr redox cycling was only 0.1% to 1% PAL (approximately 0.02% to 0.2% O_2_) [2, 3], much lower than the AOL during the Lower Carboniferous. Thus, Cr redox cycling could not be inhibited at that time, and the weathering product Cr(VI) should have transferred to the oceans and should be recorded in the carbonates.

The ~0.25 Ga Lower Triassic sample XK127 in the Daye formation

Sample XK127 is a limestone collected from the upper part of the Daye formation at Olenekian period in the Xiakou section, located 6 km east of Xiakou Town, Hubei Province, South China. The Xiakou section was deposited in a carbonate ramp across the Permian-Triassic boundary. Previous studies have suggested that the AOL reached a maximum value of ~30% O_2_ during the Permian and Triassic [15], much higher than the theoretical calculation of the minimum level of O_2_ needed to oxidize Cr(III) [2, 3]. Nevertheless, a great deal of geochemical evidence has indicated that the ocean was widely anoxic during the end of the Permian and the Lower Triassic, and the anoxic layer might have impinged onto continental shelves [16]. Considering that the Xiakou section deposited in the carbonate ramp, the ocean chemistry might not have been sufficiently oxic to stabilize Cr(VI).

The ~0.83 Ma Quaternary sample 1460A26F1W110/116 from IODP site U1460

Sample 1460A26F1W110/116 is a limestone from International Ocean Discovery Program (IODP) Site U1460 in the northern part of the Perth Basin west of Australia [17]. The sample was located ~110 m below the seafloor, and the deposition age was calculated to be ~0.83 Ma based on the age–depth model [17]. Atmospheric and oceanic environments were relatively stable during Quaternary, such that Cr cycling should be similar to that in the modern environment. Thus, this sample could serve as an analogue to modern carbonates.

**Supplementary text**

Evaluating post-depositional alteration to the carbonate samples

*Hydrothermal contamination*

The Cr concentration of hydrothermal fluids is much higher than that of seawater [18]; therefore, Cr species might be significantly influenced if carbonates are altered by hydrothermal fluids. High-temperature hydrothermal fluid is often characterized by a significant positive Eu anomaly in the REE pattern [19]; although some low-temperature hydrothermal fluids may have no Eu anomalies [20]. Therefore, the Eu anomaly of carbonates can help distinguish some types of hydrothermal contamination.

The REE+Y patterns of the carbonate samples in this study are shown in Supplementary Fig. 1, and the data were normalized to Post-Archaean Australian Shale (PAAS) [21]. The Eu anomaly was calculated using the following formula [22]:

$$\frac{Eu}{{Eu}^{*}}=\frac{Eu}{\left( {Sm}^{2}\times Tb \right)^{1/3}}$$

where *Eu*, *Sm*, and *Tb* represent the normalized weight concentration of the elements. The Eu anomalies of our samples ranged from 0.88 to 1.24 (Supplementary Table 1); thus, no Eu anomaly was observed in these samples, likely eliminating the influence of contamination from most hydrothermal fluids.

*Diagenetic alteration*

Diagenetic fluids can affect the chemical compositions of carbonates via different mechanisms. The Mn/Sr ratio is useful for screening samples for the influence of meteoric fluids because freshwater contains more Mn than seawater, and Sr is easily washed off from carbonates, whereas Mn tends to incorporate into the crystal lattice [23]. It has been suggested that carbonates with Mn/Sr < 2–3 could preserve the primary Sr isotope signals [24, 25], whereas for δ^13^C, the limitation broadens to Mn/Sr < 10 because C is relatively immobile [23].

In this study, Mn/Sr < 1.5 for all carbonates except for the Ediacaran sample [WJ705.6 (~0.63 Ga)] from the Wangji section (Supplementary Table 1). Thus, most of these samples are expected to have experienced the least diagenesis, although the exact influence of diagenetic fluids on Cr species in carbonates is poorly understood. With regard to WJ705.6 (~0.63 Ga), we note that Neoproterozoic carbonates tend to be enriched in Mn [26]; thus, Mn/Sr might be unsuitable for indicating the extent of diagenetic alteration in this case. The oxygen isotopic composition of carbonates is another proxy for diagenesis because it is very easily altered by diagenetic fluids, which are characterized by extremely light δ^18^O (–10‰ or less) [23]. The oxygen isotopic composition of Neoproterozoic seawater is thought to range between –5‰ and 0‰ [23]. The δ^18^O value of the sample [WJ705.6 (~0.63 Ga)] was –4.067‰, so this sample was unlikely to be altered by diagenesis.

Potential detrital contamination to the authigenic Cr during sample dissolution

The flatness of the REE+Y patterns of some carbonates (e.g. 12JLW44, XK127, Supplementary Fig. 1) may indicate detrital contamination. As Cr concentration in the detritus is much higher than that of the carbonate fraction, it is necessary to evaluate the influence of detrital contamination during acetic acid leaching. Therefore, we conducted a test dissolution experiment on a shale sample powder, which should simulate the detrital fraction of carbonates (e.g. silicate, organic matter), and the result showed that our sample dissolution procedure using 5% acetic acid can only dissolve ~1% of the total Cr in the shale sample. Our measured Cr concentrations in residues are no more than ~7 times to Cr concentrations in leachates, thus ~1% dissolution of Cr in residues could contribute to less than ~7% of the measured Cr concentration in carbonate fraction. This indicates that Cr is not sensitive to the detrital contamination compared to the REE during carbonate leaching.

Besides, we measured the aluminum (Al) and thorium (Th) concentrations in the leachates, two commonly used indexes of detrital contribution. The results showed that none of the samples contains significantly elevated Al or Th concentration (Supplementary Table 1), therefore suggesting limited contribution from detrital contamination during acetic acid leaching.

In the aspect of potential isotopic fractionation during leaching, if significant fractionation is associated with small dissolution of Cr in silicate, the final isotopic composition might be biased towards this fractionation. Acetate promoted dissolution of Cr(III) could cause isotopic fractionation (the maximum value for Δ^53^Cr is ~1.3‰) [27]. The largest Cr_residue_/Cr_carbonate_ of our samples is ~7, if we assume ~1% of silicate Cr dissolution (the value we determined from our shale leaching experiment), the largest isotopic bias should be ~0.09‰, which is only about twice of the analytical uncertainty, insignificant to the Cr isotopic fractionation observed in our samples.


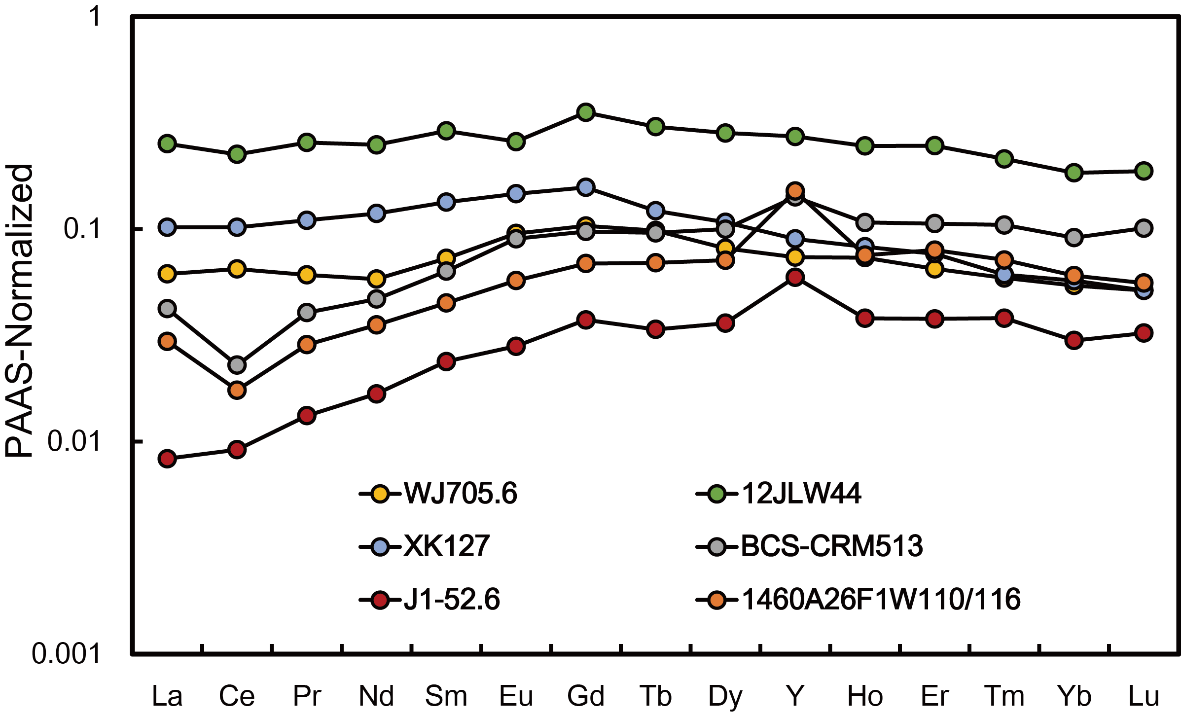


**Supplementary Figure 1:** Post-Archean Australian Shale (PAAS)-normalized REE+Y patterns of the carbonate samples.

**Supplementary Table 1: Geochemical data for the carbonate samples**

| Sample | WJ705.6 | 12JLW44 | XK127 | BCS-CRM513 | 1460A26F  1W110/116 | J1-52.6 |
| --- | --- | --- | --- | --- | --- | --- |
| Age | ~0.63 Ga | ~0.56 Ga | ~0.25 Ga | ~0.35 Ga | ~0.83 Ma | ~1.44 Ga |
| CaCO_3_ fraction (%)^a^ | 82.4 | 79.4 | 94.7 | 99.8 | 95.0 | 99.4 |
| TOC (%) | 0.03 | 0.07 | 0.04 | 0.01 | 0.30 | 0.04 |
| Mg/Ca^b^ | 0.55 | 0.05 | 0.01 | 0.01 | 0.05 | 0.59 |
| Mn/Sr^b^ | 24.0 | 0.79 | 0.07 | 0.71 | 0.01 | 1.49 |
| Eu/Eu* | 1.18 | 0.88 | 1.13 | 1.24 | 1.10 | 1.05 |
| *(wt%)*^b^ |  |  |  |  |  |  |
| Mg | 9.16 | 1.46 | 0.30 | 0.26 | 1.63 | 12.1 |
| Ca | 16.8 | 27.7 | 33.4 | 36.5 | 33.1 | 20.5 |
| Fe | 0.72 | 0.49 | 0.36 | 0.17 | 0.20 | 0.18 |
| *(ppm)*^b^ |  |  |  |  |  |  |
| Li | 2.94 | 0.65 | 0.33 | 0.22 | 0.89 | 1.26 |
| Be | 0.56 | 0.33 | 0.11 | 0.03 | 0.04 | 0.16 |
| Al | 285 | 275 | 325 | 20.0 | 38.7 | 155 |
| Sc | 2.42 | 2.56 | 1.48 | 0.27 | 0.11 | 0.11 |
| V | 4.49 | 61.9 | 2.24 | 1.21 | 2.40 | 0.84 |
| Mn | 1948 | 152 | 189 | 99.3 | 12.7 | 106 |
| Ni | 5.54 | 6.46 | 6.11 | 6.57 | 8.83 | 3.29 |
| Cu | 0.66 | 0.54 | 0.59 | 0.49 | 0.16 | 0.07 |
| Zn | 9.27 | 16.8 | 1.70 | 9.84 | 1.02 | 1.44 |
| Ga | 0.08 | 0.12 | 0.06 | 0.01 | 0.00 | 0.01 |
| Rb | 1.22 | 1.53 | 0.45 | 0.20 | 0.20 | 0.16 |
| Sr | 81.3 | 191 | 2838 | 139 | 1581 | 71.2 |
| Y | 1.99 | 7.36 | 2.43 | 3.82 | 4.08 | 1.60 |
| Zr | 0.10 | 0.13 | 0.09 | 0.14 | 0.09 | 0.17 |
| Cs | 0.01 | 0.05 | 0.01 | 0.02 | 0.00 | 0.00 |
| Ba | 5.83 | 125 | 8.30 | 95.2 | 2.84 | 3.11 |
| La | 2.35 | 9.63 | 3.89 | 1.61 | 1.13 | 0.32 |
| Ce | 5.17 | 17.9 | 8.11 | 1.82 | 1.39 | 0.73 |
| Pr | 0.54 | 2.25 | 0.97 | 0.36 | 0.25 | 0.12 |
| Nd | 1.97 | 8.44 | 4.01 | 1.59 | 1.20 | 0.57 |
| Sm | 0.40 | 1.61 | 0.74 | 0.35 | 0.25 | 0.13 |
| Eu | 0.10 | 0.28 | 0.16 | 0.10 | 0.06 | 0.03 |
| Gd | 0.48 | 1.65 | 0.73 | 0.45 | 0.32 | 0.17 |
| Tb | 0.08 | 0.23 | 0.09 | 0.07 | 0.05 | 0.03 |
| Dy | 0.38 | 1.32 | 0.50 | 0.47 | 0.33 | 0.17 |
| Ho | 0.07 | 0.24 | 0.08 | 0.11 | 0.07 | 0.04 |
| Er | 0.19 | 0.70 | 0.22 | 0.30 | 0.23 | 0.11 |
| Tm | 0.02 | 0.09 | 0.02 | 0.04 | 0.03 | 0.02 |
| Yb | 0.15 | 0.52 | 0.16 | 0.26 | 0.17 | 0.08 |
| Lu | 0.02 | 0.08 | 0.02 | 0.04 | 0.02 | 0.01 |
| Hf | 0.03 | 0.01 | 0.00 | 0.01 | 0.00 | 0.00 |
| Pb | 1.41 | 1.81 | 0.44 | 10.5 | 0.22 | 0.32 |
| Th | 0.18 | 2.09 | 0.36 | 0.08 | 0.09 | 0.06 |
| U | 0.07 | 0.26 | 1.30 | 0.48 | 4.26 | 0.13 |

^a^ Expressed as weight percentage of acid soluble fraction to the whole sample.

^b^ Concentration or weight ratio of elements in the carbonate fraction. Determined by solution ICP-MS. This fraction was leached by 5% acetic acid.

SI References:

1. Su, W, Li, H, Huff, W*, et al.* SHRIMP U-Pb dating for a K-bentonite bed in the Tieling Formation, North China. *Chin Sci Bull* 2010; **55**(29): 3312-23.

2. Planavsky, NJ, Reinhard, CT, Wang, X*, et al.* Low Mid-Proterozoic atmospheric oxygen levels and the delayed rise of animals. *Science* 2014; **346**(6209): 635-8.

3. Canfield, DE, Zhang, S, Frank, AB*, et al.* Highly fractionated chromium isotopes in Mesoproterozoic-aged shales and atmospheric oxygen. *Nat Commun* 2018; **9**(1): 2871.

4. Zhang, S, Wang, X, Wang, H*, et al.* Sufficient oxygen for animal respiration 1,400 million years ago. *Proc Natl Acad Sci U S A* 2016; **113**(7): 1731-6.

5. Planavsky, NJ, Cole, DB, Reinhard, CT*, et al.* No evidence for high atmospheric oxygen levels 1,400 million years ago. *Proc Natl Acad Sci U S A* 2016; **113**(19): E2550-1.

6. Frei, R, Gaucher, C, Poulton, SW*, et al.* Fluctuations in Precambrian atmospheric oxygenation recorded by chromium isotopes. *Nature* 2009; **461**(7261): 250-3.

7. McFadden, KA, Huang, J, Chu, X*, et al.* Pulsed oxidation and biological evolution in the Ediacaran Doushantuo Formation. *Proc Natl Acad Sci U S A* 2008; **105**(9): 3197-202.

8. Condon, D, Zhu, M, Bowring, S*, et al.* U-Pb ages from the neoproterozoic Doushantuo Formation, China. *Science* 2005; **308**(5718): 95-8.

9. Gong, Z, Kodama, KP, Li, Y-X. Rock magnetic cyclostratigraphy of the Doushantuo Formation, South China and its implications for the duration of the Shuram carbon isotope excursion. *Precambrian Res* 2017; **289**: 62-74.

10. Gilleaudeau, GJ, Frei, R, Kaufman, AJ*, et al.* Oxygenation of the mid-Proterozoic atmosphere: clues from chromium isotopes in carbonates. *Geochem Perspect Lett* 2016: 178-87.

11. Li, C, Love, GD, Lyons, TW*, et al.* A stratified redox model for the Ediacaran ocean. *Science* 2010; **328**(5974): 80-3.

12. Gao, Y, Zhang, X, Zhang, G*, et al.* Ediacaran negative C-isotopic excursions associated with phosphogenic events: Evidence from South China. *Precambrian Res* 2018; **307**: 218-28.

13. Walkden, G. The mineralogy and origin of interbedded clay wayboards in the Lower Carboniferous of the Derbyshire Dome. *Geol J* 1972; **8**(1): 143-60.

14. Lyons, TW, Reinhard, CT, Planavsky, NJ. The rise of oxygen in Earth's early ocean and atmosphere. *Nature* 2014; **506**(7488): 307-15.

15. Krause, AJ, Mills, BJW, Zhang, S*, et al.* Stepwise oxygenation of the Paleozoic atmosphere. *Nat Commun* 2018; **9**(1): 4081.

16. Lau, KV, Maher, K, Altiner, D*, et al.* Marine anoxia and delayed Earth system recovery after the end-Permian extinction. *Proc Natl Acad Sci U S A* 2016; **113**(9): 2360-5.

17. Gallagher, S, Fulthorpe, C, Bogus, K*, et al*. Expedition 356 summary. *International Ocean Discovery Program Scientific Proceedings* 2017; **356**.

18. Sander, S, Koschinsky, A. Onboard-ship redox speciation of chromium in diffuse hydrothermal fluids from the North Fiji Basin. *Mar Chem* 2000; **71**(1): 83-102.

19. Tostevin, R, Shields, GA, Tarbuck, GM*, et al.* Effective use of cerium anomalies as a redox proxy in carbonate-dominated marine settings. *Chem Geol* 2016; **438**: 146-62.

20. Bau, M. Rare-earth element mobility during hydrothermal and metamorphic fluid-rock interaction and the significance of the oxidation state of europium. *Chem Geol* 1991; **93**(3-4): 219-30.

21. Taylor, SR, McLennan, SM. *The continental crust: its composition and evolution*. Blackwell, Oxford, 1985.

22. Lawrence, MG, Greig, A, Collerson, KD*, et al.* Rare Earth Element and Yttrium Variability in South East Queensland Waterways. *Aquat Geochem* 2006; **12**(1): 39-72.

23. Kaufman, AJ, Knoll, AH. Neoproterozoic variations in the C-isotopic composition of seawater: stratigraphic and biogeochemical implications. *Precambrian Res* 1995; **73**(1): 27-49.

24. Derry, LA, Kaufman, AJ, Jacobsen, SB. Sedimentary cycling and environmental change in the Late Proterozoic: evidence from stable and radiogenic isotopes. *Geochim Cosmochim Acta* 1992; **56**(3): 1317-29.

25. Kaufman, AJ, Jacobsen, SB, Knoll, AH. The Vendian record of Sr and C isotopic variations in seawater: implications for tectonics and paleoclimate. *Earth Planet Sci Lett* 1993; **120**(3): 409-30.

26. Herrington, PM, Fairchild, IJ. Carbonate shelf and slope facies evolution prior to Vendian glaciation, central East Greenland. *The Caledonide Geology of Scandinavia* 1989: 263-73.

27. Saad, EM, Wang, X, Planavsky, NJ*, et al.* Redox-independent chromium isotope fractionation induced by ligand-promoted dissolution. *Nat Commun* 2017; **8**(1): 1590.
